# The Risk Factors for Chinese Medical Economic Burden of Aging: A Cross-Sectional Study Based on Guangdong Province

**DOI:** 10.1155/2021/6680441

**Published:** 2021-07-07

**Authors:** Jialong Chen, Zhenzhu Qian, Liuna Yang, Ting Liu, Mingwei Sun, Honglin Yu, Chonghua Wan, Yunbin Yang

**Affiliations:** ^1^School of Public Health, Guangdong Medical University, Dongguan 523808, China; ^2^Dongkeng Town Center for Disease Control and Prevention, Dongguan City 523808, China; ^3^School of Humanities and Management, Research Center for Quality of Life and Applied Psychology, Guangdong Medical University, Dongguan 523808, China; ^4^Guangdong Medical University, Dongguan 523808, China

## Abstract

**Background:**

The proportion of aging in China is increasing, which needs more healthcare recourses. To analyze the risk factors of the direct medical economic burden of aging in China and provide the strategies to control the cost of treatment, the information was collected based on Guangdong Province's regular health expenditure accounting data collection plan.

**Methods:**

The multiple linear regression models were used to explore the risk factors of inpatient expenses of the elderly in Guangdong province.

**Results:**

The results revealed that hospital day, age, male patients, and patients who suffer from malignant tumors are key factors to increase the direct medical economic burden of aging. Moreover, the medical insurance for urban employees can reduce the medical economic burden, comparing with the medical insurance for urban residents.

**Conclusions:**

The basic medical insurance system and the serious illness insurance system should be improved. While striving to speed up the development of regional economy, the government should pay attention to the construction of basic medical institutions in economically backward areas, increase the allocation of health human resources, and facilitate the masses to seek medical treatment nearby.

## 1. Background

The 21st century is an era of global population aging that has become a global long-term and universal topic of concern. There are 76 countries (regions) with aging population in 2010, which is expected to increase to 157 in 2050. There is no doubt that China faces great pressure of aging. According to the data of 1998 population sampling survey (sampling ratio 1.01‰) published in China Statistical Yearbook 1999, the population aged over 65 accounts for 7.43% of the total population, which shows the trend of aging. According the Basic China Statistical Yearbook 2019, the population aged over 65 reached 160 million, accounting for 11.9% of the total population. After the 1980s, China has formed a trend of low fertility rate and reduction in the size of the birth population and further strengthened the changing trend of the population age structure, so that the proportion of the elderly population will exceed 30% in the future and will continue to be high. The process of population growth is also the process of population aging [1]. The proportion of aging in China is increasing, which needs more healthcare recourses. The physical quality of the elderly weakens with age, and the chance of suffering from various diseases increases accordingly, especially chronic noncommunicable diseases [2]. This study is aimed at analyzing the main risk factors that cause the medical economic burden of the elderly and at proposing policy recommendations. In this work, we found that the per capita cost of the age group over 60 years old, the per capita cost of hospitalization, and the per capita cost of large outpatient clinics are significantly higher than those of other age groups. The aging of the population has very obvious relevance to medical expenses. This effect occurs earlier in my country than in developed countries [3].

A study found that the out of pocket expenses of the elderly accounted for 72.38% of the total medical expenses in 2015, while the personal health expenses of the whole population accounted for 29.27% of the total health expenses according to the China Health Statistics Yearbook, indicating that the medical economic burden of the elderly is far higher than that of other groups [4]. Therefore, we should pay attention to improve the health status of the elderly and reduce the medical and economic burden of the elderly.

There are many studies on medical economic burden, which mainly focuses on the analysis of single disease and scattered areas and lacking the comparative analysis of aged population [5–7]. From the perspective of health service fairness, this study analyzes the difference of the proportion of health care expenditure to per capita consumption expenditure of residents in Guangdong Province. This study discusses the influence factors of the elderly residential expenses. This work will conduct an empirical analysis of the factors affecting the medical expenses of the elderly population, give scientific enlightenment to the elderly to reduce medical expenses, and provide a policy reference for the government to ease the medical expenses of the elderly population. Theory not only can enrich the relevant research content but also is conducive to improving the health level of residents in various regions and reducing their economic burden, which is of great theoretical and practical significance.

## 2. Methods

### 2.1. Study Design

This is a cross-sectional study. According to the 2016 regular health expenditure accounting data collection plan of Guangdong Province, multistage stratified random sampling (City District County Street Township Community Village) was conducted to collect the outpatient and inpatient information of various medical institutions in 2016, including age, gender, hospital day, disease name, expense information, insurance type, financing source, institution type, and institution level. The data was cleaned and standardized, and the database was established after removing the information of patients under 65 years old.

### 2.2. Participants

In this study, 1184563 cases of elderly patients over 65 years old in Foshan and Shanwei in 2016 were collected, and 1081980 cases (928338 cases in Foshan and 153642 cases in Shanwei) were effectively recorded, and the effective rate was 91.34%.

### 2.3. Variables

Age, gender, hospital day, disease name, expense information, insurance type, financing source, institution type, and institution level are the variables used in the study.

### 2.4. Bias

There may be selection bias and sampling bias. The findings from this brief cross-sectional study are only suggestive (not confirmative) for causal associations.

### 2.5. Study Size

The sample size calculation was basic on the Sample size calculation formula: *n* = (*tαS*/*δ*)^2^, where *S* is an estimate of the population standard deviation, *δ* = 0.1, and *α* = 0.05. *Z*^*α*^/2(1.96) is used to substitute the formula and calculate *n*. Then, calculate the initial degrees of freedom, and substitute this *t* into the formula to get the second estimated *n*. Iterate in this way until *n* is stable. Taking into account the large sampling range and the large degree of sample variation, we have further expanded the sample size on the basis of the calculation formula. In this study, 1184563 cases of elderly patients over 65 years old in Foshan and Shanwei in 2016 were collected, and 1081980 cases (928338 cases in Foshan and 153642 cases in Shanwei) were effectively recorded, and the effective rate was 91.34%. The research scope of the project is Guangdong Province, and Foshan and Shanwei are selected as the research cities in Guangdong Province, which represent the economically developed and underdeveloped regions of Guangdong Province, respectively. In this study, 1184563 cases of elderly patients over 65 years old in Foshan and Shanwei in 2016 were collected, and 1081980 cases (928338 cases in Foshan and 153642 cases in Shanwei) were effectively recorded, and the effective rate was 91.34%.

### 2.6. Statistical Methods

SAS 9.4 statistical software was used to analyze the influence factors of inpatient expenses with a multiple linear regression model. Age, gender, length of stay, name of disease, type of medical insurance, heavy economic burden or not, and type of hospital were covariates. For the classified variables, they are first converted to dummy variables (dummy variables) and then introduced into the model. The difference was statistically significant (*P* < 0.05).

### 2.7. Data Collection

The data was cleaned and standardized, and the database was established after removing the information of patients under 65 years old. The detailed sampling process is as follows.

In the first phase, Guangdong Province was divided into the Pearl River Delta region (Guangzhou, Shenzhen, Zhuhai, Foshan, Jiangmen, Dongguan, Zhongshan, Huizhou, and Zhaoqing) and non-Pearl River Delta region (Shantou, Shaoguan, Heyuan, Meizhou, Shanwei, Yangjiang, Zhanjiang, Maoming, Qingyuan, Chaozhou, Jieyang, and Yunfu) based on economic levels. One city was randomly selected from the Pearl River Delta region, while the other one was randomly selected from the non-Pearl River Delta region. Finally, Foshan and Shanwei were selected. City-level sample institutions include city-level maternal and child health centers and city-level general hospitals, city-level Chinese medicine hospitals, and various specialized hospitals in the two cities.

In the second stage, three districts or counties are selected from each of the two cities. Nanhai District, Sanshui District, and Gaoming District were selected in Foshan City. Urban District, Haifeng County, and Luhe County are selected as the counties in Shanwei City. One general hospital, maternal and child health care hospital, and traditional Chinese medicine hospital were selected from each district and county.

In the third stage, 5-8 community health service centers and hospitals were selected for each district and county, and 6 community health service stations and 6 village health rooms were selected for each community health service center. One to three outpatient departments were selected in each district and county according to the number of affiliated outpatient departments, and 5-20 individual clinics were selected according to the number of affiliated clinics.

### 2.8. Data Quality Control

The data cleaning personnel were trained to clean and standardize the data according to the variable standardization comparison table. By using VLOOKUP function of Excel 2013 software, the disease name was standardized and coded according to the international classification of Diseases 10th Revision (ICD-10). Error information was deleted, and then Stata 12.0 software was used to establish the database.

## 3. Results

### 3.1. Basic Characteristics of Data

In this study, 1184563 cases of elderly patients over 65 years old in Foshan and Shanwei in 2016 were collected, and 1081980 cases (928338 cases in Foshan and 153642 cases in Shanwei) were effectively recorded, and the effective rate was 91.34%; the hospitalization records were 57553 cases, and the effective records were 51873 cases (44510 cases in Foshan and 7363 cases in Shanwei), and the effective rate was 90.13%. In terms of the distribution of elderly outpatients in the two cities, women accounted for 55.04%, and men accounted for 44.96%; in terms of age distribution, the highest proportion was the 65-74 age group, accounting for 60.86%; in terms of types of insurance, urban residents accounted for 52.56%; in terms of medical institutions, primary medical and health institutions accounted for the largest proportion, accounting for 49.61%. In terms of the distribution of elderly inpatients in the two cities, women accounted for 50.23%, and men accounted for 49.77%; in terms of age distribution, the highest proportion was 65-74 age group, accounting for 48.10%; in terms of types of insurance, urban residents accounted for 66.58%; in terms of medical institutions, TCM hospitals accounted for the largest proportion, accounting for 48.01% (Table 1).

### 3.2. The Risk Factors on the Total Hospitalization Cost of over 65 Years Old

The multiple linear regression was established; it can be seen from Table 1 indicating that the regression model is effective (*P* < 0.001). The total cost of hospitalization is affected by the hospital day, age, sex, malignant tumor, respiratory system diseases, nutrition and metabolism diseases, digestive system diseases, medical insurance for urban employees, medical insurance for urban residents, and heavy economic burden.

The results shown that the total hospitalization cost is increased by 134.04 USD (867.60 RMB) for each additional hospital day. The average total cost of hospitalization is increased by 14.69 USD (95.10 RMB) for each additional year of age. In terms of gender, the total cost of hospitalization for male patients is 104.84 USD (678.56 RMB) more than that for female patients. For the variable of disease type, the total cost of patient suffering from malignant tumors is increased by 511.90 USD (3313.29 RMB) on average. The total cost of hospitalization of patient suffering from respiratory system diseases is increased by 233.27 USD (1509.81 RMB) on average. Patient suffers from nutrition and metabolic diseases and digestive system diseases; the total cost of hospitalization is decreased by 527.08 USD (3411.50 RMB) and 255.70 USD (1654.99 RMB) on average. For the types of insurance, if the patients participate in the medical insurance for urban employees, the total cost of hospitalization is reduced by 320.20 USD (2072.47 RMB) on average. If a patient participates in the medical insurance for urban residents, the total cost of hospitalization is reduced by 216.61 USD (1401.99 RMB) on average. Moreover, if a patient belongs to the case with heavy economic burden, the total cost of hospitalization is increased by an average of 901.45 USD (5834.65 RMB) (Table 2).

### 3.3. The Risk Factors on the Treat Cost of Hospitalization of over 65 Years Old

The multiple linear regression was established; it can be seen from Table 3 indicating that the regression model is effective (*P* < 0.001). The hospital day, age, gender, malignant tumors, respiratory diseases, nutritional and metabolic diseases, digestive system diseases, urban employee medical insurance, urban resident medical insurance, and self-payment have impact on the cost of hospitalization.

Results reveal that the cost of the treatments is increased by an average of 25.90 USD (167.63 RMB) for each additional hospital day, and the cost of the treatments is increased by 6.04 USD (39.09 RMB) for each additional year of age. In terms of gender, the cost of the treatments of males is 30.83 USD (199.53 RMB) more than that of females. For the variable of disease type, in a patient suffering from a malignant tumor, the cost of the treatments is decreased by an average of 212.11 USD (1372.86 RMB). If the patient suffers from respiratory disease, the cost of the treatments is decreases by an average of 60.56 USD (391.99 RMB). If the patient suffers from nutrition and metabolic diseases and digestive system diseases, the cost of the treatments is decreased by an average of 154.27 USD (-898.77 RMB) and 138.86 USD (898.77 RMB). For the type of insurance, if the patient participates in urban employee medical insurance, the cost of the treatments is reduced by an average of 471.62 USD (3052.54 RMB). If the patient participates in urban resident's medical insurance, the cost of the treatments is reduced by an average of 393.00 USD (2543.69 RMB). Interestingly, if the patient is a fully self-payment case, the cost of hospitalization is decreased by an average of 348.22 USD (2253.85RMB) (Table 3).

### 3.4. The Risk Factors on the Inpatient Examination Fee of over 65 Years Old

The multiple linear regression was established; it can be seen from Table 4 indicating that the regression model is effective (*P* < 0.001). The factors that affect the cost of inpatient examination are hospital day, age, gender, circulatory system disease, respiratory system disease, digestive system disease, medical insurance for urban employees, medical insurance for urban residents, self-payment, heavy economic burden, general hospital, hospital of traditional Chinese medicine, specialized hospital, and basic medical and health institutions.

Results showed that the average increase of examination fee is 5.07 USD (32.81 RMB) for each additional hospital day and the average cost of examination is increased by 2.06 USD (13.36 RMB) for each additional year of age. Compared with that for female patients, the average cost of hospitalization examination for male patients is 18.06 USD (116.87 RMB) more. For the types of diseases, if the patient suffers from circulatory system disease, the average cost of examination is increased by 59.45 USD (384.81 RMB). If the patient suffers from respiratory system disease, the average cost of examination is increased by 56.23 USD (363.96 RMB). If the patient suffers from digestive system diseases, the average cost of examination is increased 19.54 USD (126.45 RMB). For the type of insurance, if the patient participates in the medical insurance for urban employees, the cost of examination is reduced by 37.99 USD (245.92 RMB) on average. If the patient participates in the medical insurance for urban residents, the cost of examination is reduced by 49.39 USD (319.65 RMB) on average. If the patient suffers from heavy economic burden, the average cost of hospitalization examination is increased by 12.13 USD (78.48 RMB). If the patient was treated in general hospitals, traditional Chinese medicine hospitals, specialized hospitals, and grassroots medical and health institutions, the average cost of examination is reduced by 126.99 USD (821.97 RMB), 66.22 USD (428.63 RMB),170.06 USD (1100.74 RMB), and 240.09 USD (1554.00 RMB) (Table 4).

### 3.5. The Risk Factors on the Inpatient Drug Cost of over 65 Years Old

The multiple linear regression was established; it can be seen from Table 5 below indicating that the regression model is effective (*P* < 0.001). The factors that affect the cost of inpatient drug cost are hospital day, age, gender, circulatory system diseases, malignant tumors, respiratory system diseases, digestive system diseases, and heavy economic burden.

Results showed that the average increase of hospitalization drug cost is 44.38 USD (287.28 RMB) for each additional hospital day and the average cost of drugs is increased by 5.19 USD (33.62 RMB) for each additional year of age. Compared with that of the female patient, the average cost of drugs of male is 93.02 USD (602.08 RMB) more. If the patient suffers from circulatory system diseases, the average cost of drugs is increased by 253.72 USD (1642.21 RMB). If the patient suffers from malignant tumors, the average cost of hospitalization drugs is increased by 465.99 USD (3016.09 RMB). If the patient suffers from respiratory system diseases, the average cost of hospitalization drugs is increased by 530.44 USD (3433.27 RMB). If the patient suffers from digestive system disease, the average cost of hospitalization drugs is increased by 184.01 USD (1190.98 RMB). For the patient with heavy economic burden, the cost of hospitalization drugs is increased by an average of 255.93 USD (1656.49 RMB) (Table 5).

### 3.6. The Risk Factors on the Other Expenses for Hospitalization of over 65 Years Old

The multiple linear regression was established; it can be seen from Table 6 below indicating that the regression model is effective (*P* < 0.001). The other expenses that affect hospitalization are hospital days, age, gender, circulatory system diseases, malignant tumors, respiratory system diseases, nutrition and metabolism diseases, digestive system diseases, medical insurance for urban employees, medical insurance for urban residents, self-payment, heavy economic burden, and traditional Chinese medicine hospital.

Results show that the average cost of hospitalization expenses is increased by 26.08 USD (168.79 RMB) for each additional hospital day, and the average cost of other hospitalization expenses is increased by 12.57 USD (81.34RMB) for each additional year of age. Compared with that of the female patient, the other hospitalization expenses of the male patient are 59.74 USD (386.66RMB) less. If the patient suffers from circulatory system diseases, malignant tumors, respiratory system diseases, nutrition and metabolism diseases, and digestive system diseases, the average cost of other hospitalization expenses is reduced by 387.51 USD (2508.16 RMB), 527.24 USD (3412.58 RMB), 428.30 USD (2772.20 RMB), 801.55 USD (5188.04 RMB), and 576.46 USD (3731.11 RMB). For the types of insurance, if the patient participates in urban employee medical insurance and urban resident medical insurance, the average cost of other hospitalization expenses is reduced by 907.53 USD (5874.00 RMB) and 535.75 USD (3467.64 RMB).

If the patient is a fully self-payment case, the average cost of other hospitalization expenses is reduced by 191.02 USD (1236.36 RMB). If a patient with a heavy economic burden, the average cost of other hospitalization expenses is increased by 482.99 USD (3126.16 RMB). In terms of medical institutions, if the patient was treated in the hospital of traditional Chinese medicine for medical treatment, the average cost of other hospitalization expenses is increased by 364.63 USD (2360.04 RMB) (Table 6).

## 4. Discussion

The age of patients is a risk factor for the increase of total outpatient and inpatient costs. Age can fully predict the severity of a variety of diseases, so the older the patient is, the more difficult the treatment is and the more medical resources are consumed. For example, in COVID-19 global pandemic in 2020, a number of scholars analyzed the hospitalization expenses of 105 confirmed patients in Shenzhen by multiple linear regression analysis. The results revealed that the average hospitalization cost increased by 24.12 USD for every additional year. Therefore, the higher the age, the greater the economic burden of direct medical treatment [8]. On the other hand, the proportion of malignant tumors, coronary heart disease, and other serious diseases in the elderly patient increases significantly with the increase of age, and the demand for health services is also higher [9]. There is another view in academic circles that aging causes medical expenses to rise. Scholars such as Zweifel et al. and Fen et al. deny the influence of age on medical expenses. They believe that among people aged 65 and above, the time of death is the main factor affecting medical expenses. The effect of age is not a significant factor if you ignore the effect of imminent death, as it may cause overestimation of the effect of age on medical expenses. [10, 11]. To cope with the challenge of the burden of medical expenses caused by aging, the supply of medical service resources for the elderly should be increased, and the establishment of a health and elderly care industry should be encouraged to replace medical treatment with long-term care to achieve a precise allocation of medical resources and long-term care. Besides, encourage the development of hospice care for the elderly, so that life can end with dignity.

The total hospitalization cost of male elderly patients is higher than that of female elderly patients. This is consistent with previous research conclusions. For example, Zhang et al. used the data of stroke cases from Guangzhou medical insurance claim database to establish multiple linear regression and analyzed the influencing factors of hospitalization expenses. The results showed that the average hospitalization expenses of male patients were higher than those of female patients [12]. In the study of Yang J. and Yang L., we also got the conclusion that the inpatient cost of the elderly male patients in all age groups is higher than that of the elderly female patients, which shows that gender is an important factor affecting the medical cost burden of the elderly [13]. The reasons why there is a gender difference in medical expenses are as follows: first, the incidence and severity of disease are different as the disease spectrums vary for different genders. Compared with women, men have a higher proportion for tobacco and alcohol dependence as well as unhealthy lifestyles [14–16], which have also aggravated the occurrence and development of chronic diseases in males. Second, men are under greater social and family pressure. They are more likely to be exposed to high-risk industries, and the incidence of accidental injuries is higher [17]. Third, females have fewer capabilities of financial security, resulting in lower utilization of health resources than males, where there is more difference when major diseases occur. The health management department should pay more attention to male elderly when carrying out health management work for the elderly, to effectively reduce the demand for male elderly medical services. On the other hand, improving gender equity and increasing women's social status and economic capacity can increase the utilization of female health resources. Older people with chronic diseases face a higher medical-economic risk than those without, and many previous studies have come to the same conclusion. Malignant tumors are a major risk factor for high hospitalization costs in the elderly. Malignant tumors, due to the serious illness, the large amount of drugs used throughout the course, the high drug price, the difficulty of treatment, and the long time, bring a heavy medical economic burden to the family and society, and it is easy to cause residents to become poor or give up treatment [18]. A study on the factors affecting the hospitalization costs of patients with chronic diseases in Sichuan, Zhejiang, and Tianjin showed that the degree of the impact of malignant tumors was on the top 3, which was the main risk factor for increasing the hospitalization costs [19, 20]. Circulatory system diseases and respiratory system diseases have a long course, many hospital admissions, long hospital stays, and high material costs, which also lead to high medical expenses. In response to the high medical expenditures caused by chronic diseases of the elderly, the following aspects can be taken: on the one hand, strengthen health education, extensively develop community chronic disease health self-management groups, improve the health awareness of the elderly, reduce the incidence of chronic diseases, and delay chronic venereal diseases. On the other hand, formulate reasonable policies and preferential policies to broaden the benefits of primary medical facilities, guide mild patients to primary medical institutions for treatment, reduce excessive waste of medical resources in tertiary hospitals, and realize the rational allocation of health resources.

Different insurance types have an impact on hospitalization costs. This conclusion is consistent with multiple studies. Studies have shown that the outpatient consultation rate and inpatient service utilization rate of insured persons are higher than those of noninsured persons; for every 10% increase in the outpatient reimbursement rate, the outpatient cost per capita increases by 7.7%; for every 10% increase in the inpatient reimbursement rate, the per capita hospitalization costs up to 3.82% [21]. Xiong et al.'s research shows that after China established the universal medical insurance system in 2008, the catastrophic expenditure rate of urban employees' basic medical insurance inpatients is the lowest among the inpatients with different income levels [22]. Another project is to compare the direct medical costs of inpatients with different types of medical insurance for ischemic stroke in China. The per capita hospitalization cost of urban employees' medical insurance is 1565.24 USD, the per capita cost is 362.46 USD, and the reimbursement rate is 76.84%. The per capita hospitalization cost of urban residents' medical insurance is 1183.78 USD. The per capita self-pay is 518.50 dollars, and the reimbursement rate is 56.20%. The hospitalization cost of urban employees' medical insurance patients is higher than that of urban residents' medical insurance patients, but the self-financed amount and the proportion of the self-financed amount in the total cost are lower than the latter, so the medical financial burden is also lower than the latter [23]. In addition, Ding et al. also believe that different types of medical insurance have different effects on hospitalization costs [24]. Generally, compared with other types of medical insurance, the basic medical insurance for urban employees can provide a higher reimbursement ratio for beneficiaries, and the actual cost is lower than other types of medical insurance [25]. Social demographic factors such as income and education level of urban employee medical insurance participants are better than those of urban residents' medical insurance and rural cooperative medical insurance participants, so the emphasis on health and willingness to use medical services are higher than others. This is also one of the reasons for the higher medical expenses of urban employee medical insurance participants. Because of the differences in medical expenses caused by different insured situations, the author believes that it can expand the coverage of medical insurance, consolidate and improve the new rural cooperative medical system and the urban residents' medical insurance system, improve the medical insurance protection standards, and encourage the elderly with better economic conditions to participate. Commercial medical insurance can comprehensively improve the affordability of medical expenses for the elderly.

The increase in the length of hospitalization will significantly increase the cost of hospitalization. This conclusion is consistent with the conclusions of multiple studies [26–28]. For example, a study conducted in the United States showed that shortening the length of hospital stay is one of the main ways to reduce the cost of hospitalization [29]. Xiaodong established multiple linear regression on the factors related to the hospitalization costs of 1554 cases of acute ST-segment elevation myocardial infarction and found that the number of hospitalization days had the greatest impact on hospitalization costs [30]. Generally, the more hospitalization days, the more serious the patient's condition or the need for long-term treatment, and the more resources consumed, so the higher the hospitalization cost.

This study may have some limitations. The income level has a very important influence on medical expenses. Influencing factors such as insurance type and gender are closely related to the income level. This study did not thoroughly explore the relationship between the income level of the elderly and medical expenses. This aspect needs further research.

## Figures and Tables

**Table 1:** basic information of inpatient sample data.

Variable		Foshan City		Shanwei City		Total	
		Number of cases	Proportion (%)	Number of cases	Proportion (%)	Number of cases	Proportion (%)
Gender	Male	21500	48.30	4316	58.62	25816	49.77
	Female	23010	51.70	3047	41.38	26057	50.23

Age (years)	65-74	21877	49.15	3076	41.78	24953	48.10
	75-84	16777	37.69	3318	45.06	20095	38.74
	Over 85	5856	13.16	969	13.16	6825	13.16

Type of insurance	Urban workers	8273	18.59	86	1.17	8359	16.11
	Urban residents	27394	61.55	7144	97.03	34538	66.58
	Self-paid	7105	15.96	104	1.41	7209	13.90
	Other types of insurance	1738	3.90	29	0.39	1767	3.41

Types of medical institutions	General hospital	16198	36.39	3535	48.01	19733	38.04
	Chinese medicine hospital	24096	54.14	810	11.00	24906	48.01
	Specialized hospital	2721	6.11	0	0.00	2721	5.25
	Primary-level medical and health care institutions	1433	3.22	3018	40.99	4451	8.58
	Maternal and child health hospital	62	0.14	0	0.00	62	0.12
	Outpatient department	0	0.00	0	0.00	0	0.00

**Table 2:** The risk factors on the total hospitalization cost of over 65 years old.

Variable	*β* coefficient	Standard error	95% CI		*t*	Pr > ∣*t*∣
Intercept	-5490.00	3907.39	-13148.4844,	2168.4844	-1.41	0.160
Hospital day	867.60	11.47	845.1188	890.0812	75.63	<0.001
Age	95.10	18.65	58.546	131.654	5.10	<0.001
Gender	678.56	275.26	139.0504	1218.0696	2.47	0.014
Circulatory diseases	625.41	354.18	-68.7828	1319.6028	1.77	0.077
Malignant tumors	3313.29	608.48	2120.6692	4505.9108	5.45	<0.001
Respiratory system diseases	1509.81	432.19	662.7176	2356.9024	3.49	<0.001
Nutrition and metabolic diseases	-3411.50	589.71	-4567.3316	-2255.6684	-5.79	<0.001
Digestive system diseases	-1654.99	508.40	-2651.454	-658.526	-3.26	0.001
Other diseases	0.00	.	.	.	.	.
Urban employees insurance	-2072.47	563.17	-3176.2832	-968.6568	-3.68	<0.001
Urban resident insurance	-1401.99	466.63	-2316.5848	-487.3952	-3.00	0.003
New rural cooperative medical system	2518.54	2223.47	-1839.4612	6876.5412	1.13	0.257
Commercial insurances	1165.74	737.93	-280.6028	2612.0828	1.58	0.114
Self-payment	-932.99	600.45	-2109.872	243.892	-1.55	0.120
Other insurances	0.00	.	.	.	.	.
Heavy economic burden	5834.65	339.55	5169.132	6500.168	17.18	<0.001
General hospital	1285.22	3612.26	-5794.8096	8365.2496	0.36	0.722
Chinese medicine hospital	-29.61	1807.36	-3572.0356	3512.8156	-0.02	0.987
Specialized hospital	-8243.20	3654.35	-15405.726	-1080.674	-2.26	0.024
Primary medical and health institutions	-6015.50	3637.88	-13145.7448	1114.7448	-1.65	0.098
Maternal and child health hospital	0.00	.	.	.	.	.

Note: *F* = 432.320. ^∗∗∗^*P* < 0.001.

**Table 3:** The risk factors on the cost of hospitalization of over 65 years old.

Variable	*β* coefficient	Standard error	95% CI		*t*	Pr > ∣*t*∣
Intercept	-805.13	1029.54	-2823.0284	1212.7684	-0.78	0.434
Hospital day	167.63	3.02	161.7108	173.5492	55.43	<0.001
Age	39.09	4.96	29.3684	48.8116	7.88	<0.001
Gender	199.53	73.22	56.0188	343.0412	2.73	0.006
Circulatory diseases	-122.49	93.99	-306.7104	61.7304	-1.30	0.193
Malignant tumors	-1372.86	161.10	-1688.616	-1057.104	-8.52	<0.001
Respiratory system diseases	-391.99	116.30	-619.938	-164.042	-3.37	<0.001
Nutrition and metabolic diseases	-998.50	155.78	-1303.8288	-693.1712	-6.41	<0.001
Digestive system diseases	-898.77	135.43	-1164.2128	-633.3272	-6.64	<0.001
Other diseases	0.00	.	.	.	.	.
Urban employee insurance	-3052.54	150.75	-3348.01	-2757.07	-20.25	<0.001
Urban resident insurance	-2543.69	126.45	-2791.532	-2295.848	-20.12	<0.001
New rural cooperative medical system	555.73	574.59	-570.4664	1681.9264	0.97	0.334
Commercial insurances	676.18	508.37	-320.2252	1672.5852	1.25	0.181
Self-payment	-2253.85	160.63	-2568.6848	-1939.0152	-14.03	<0.001
Other insurances	0.00	.	.	.	.	.
Heavy economic burden	102.26	89.77	-73.6892	278.2092	1.14	0.255
General hospital	118.84	949.36	-1741.9056	1979.5856	0.13	0.900
Chinese medicine hospital	504.16	475.01	-426.8596	1435.1796	1.06	0.289
Specialized hospital	-1139.06	960.42	-3021.4832	743.3632	-1.19	0.236
Primary medical and health institutions	-1627.46	957.46	-3504.0816	249.1616	-1.70	0.089
Maternal and child health hospital	0.00	.	.	.	.	.

Note: *F* = 245.760. ^∗∗∗^*P* < 0.001.

**Table 4:** The risk factors on the Inpatient examination fee of over 65 years old.

Variable	*β* coefficient	Standard error	95% CI		*t*	Pr > ∣*t*∣
Intercept	440.84	388.87	-321.3452	1203.0252	1.13	0.257
Hospital day	32.81	1.18	30.4972	35.1228	27.71	<0.001
Age	13.36	2.00	9.44	17.28	6.67	<0.001
Gender	116.87	29.76	58.5404	175.1996	3.93	<0.001
Circulatory diseases	384.81	37.91	310.5064	459.1136	10.15	<0.001
Malignant tumors	-2.18	79.56	-158.1176	153.7576	-0.03	0.978
Respiratory system diseases	363.96	46.78	272.2712	455.6488	7.78	<0.001
Nutrition and metabolic diseases	10.05	59.52	-106.6092	126.7092	0.17	0.866
Digestive system diseases	126.45	57.97	12.8288	240.0712	2.18	0.029
Other diseases	0.00	.	.	.	.	.
Urban employee insurance	-245.92	63.88	-371.1248	-120.7152	-3.85	<0.001
Urban resident insurance	-319.65	50.37	-418.3752	-220.9248	-6.35	<0.001
New rural cooperative medical system	380.10	253.22	-116.2112	876.4112	1.50	0.133
Commercial insurances	-40.17	84.22	-205.2412	124.9012	-0.48	0.633
Self-payment	-510.11	65.06	-637.6276	-382.5924	-7.84	<0.001
Other insurances	0.00	.	.	.	.	.
Heavy economic burden	78.48	39.85	0.374	156.586	1.97	0.049
General hospital	-821.97	354.44	-1516.6724	-127.2676	-2.32	0.020
Chinese medicine hospital	-428.63	177.27	-776.0792	-81.1808	-2.42	0.016
Specialized hospital	-1100.74	359.09	-1804.5564	-396.9236	-3.07	0.002
Primary medical and health institutions	-1554.00	358.11	-2255.8956	-852.1044	-4.34	<0.001
Maternal and child health hospital	0.00	.	.	.	.	.

Note: *F* = 73.350. ^∗∗∗^*P* < 0.001.

**Table 5:** The risk factors on the Inpatient drug cost of over 65 years old.

Variable	*β* coefficient	Standard error	95% CI		*t*	Pr > ∣*t*∣
Intercept	-4720.10	1892.43	-8429.2628	-1010.9372	-2.49	0.013
Hospital day	287.28	4.52	278.4208	296.1392	63.49	<0.001
Age	33.62	7.90	18.136	49.104	4.26	<0.001
Gender	602.08	115.38	375.9352	828.2248	5.22	<0.001
Circulatory diseases	1642.21	150.35	1347.524	1936.896	10.92	<0.001
Malignant tumors	3016.09	243.37	2539.0848	3493.0952	12.39	<0.001
Respiratory system diseases	3433.27	183.51	3073.5904	3792.9496	18.71	<0.001
Nutrition and metabolic diseases	428.81	234.39	-30.5944	888.2144	1.83	0.067
Digestive system diseases	1190.98	215.19	769.2076	1612.7524	5.53	<0.001
Other diseases	0.00	.	.	.	.	.
Urban workers	644.82	375.43	-91.0228	1380.6628	1.72	0.086
Urban residents	411.83	352.50	-279.07	1102.73	1.17	0.243
New rural cooperative medical system	343.49	1021.73	-1659.1008	2346.0808	0.34	0.737
Commercial insurances	600.55	369.14	-122.9644	1324.0644	1.63	0.104
Self-payment	-714.45	380.80	-1460.818	31.918	-1.88	0.061
Other insurances	0.00	.	.	.	.	.
Heavy economic burden	1656.49	141.89	1378.3856	1934.5944	11.67	<0.001
General hospital	233.48	1767.51	-3230.8396	3697.7996	0.13	0.895
Chinese medicine hospital	-510.63	883.83	-2242.9368	1221.6768	-0.58	0.563
Specialized hospital	-2886.42	1779.13	-6373.5148	600.6748	-1.62	0.105
Primary medical and health institutions	-595.89	1781.07	-4086.7872	2895.0072	-0.33	0.738
Maternal and child health hospital	0.00	.	.	.	.	.

Note: *F* = 296.570. ^∗∗∗^*P* < 0.001.

**Table 6:** The risk factors on the other expenses for hospitalization of over 65 years old.

Variable	*β* coefficient	Standard error	95% CI		*t*	Pr > ∣*t*∣
Intercept	-1872.62	2187.13	-6159.3948	2414.1548	-0.86	0.392
Hospital day	168.79	6.42	156.2068	181.3732	26.30	<0.001
Age	81.34	10.65	60.466	102.214	7.64	<0.001
Gender	-386.66	157.07	-694.5172	-78.8028	-2.46	0.014
Circulatory diseases	-2508.16	201.97	-2904.0212	-2112.2988	-12.42	<0.001
Malignant tumors	-3412.58	343.81	-4086.4476	-2738.7124	-9.93	<0.001
Respiratory system diseases	-2772.20	250.83	-3263.8268	-2280.5732	-11.05	<0.001
Nutrition and metabolic diseases	-5188.04	330.62	-5836.0552	-4540.0248	-15.69	<0.001
Digestive system diseases	-3731.11	290.05	-4299.608	-3162.612	-12.86	<0.001
Other diseases	0.00	.	.	.	.	.
Urban worker insurance	-5874.00	346.83	-6553.7868	-5194.2132	-16.94	<0.001
Urban resident insurance	-3467.64	298.77	-4053.2292	-2882.0508	-11.61	<0.001
New rural cooperative medical system	-110.04	1253.34	-2566.5864	2346.5064	-0.09	0.930
Commercial insurances	614.96	476.45	-318.882	1548.802	1.29	0.197
Self-payment	-1236.36	364.46	-1950.7016	-522.0184	-3.39	<0.001
Other insurances	0.00	.	.	.	.	.
Heavy economic burden	3126.16	192.46	2748.9384	3503.3816	16.24	<0.001
General hospital	-1090.46	2007.89	-5025.9244	2845.0044	-0.54	0.587
Chinese medicine hospital	2360.04	1004.64	390.9456	4329.1344	2.35	0.019
Specialized hospital	-2296.17	2031.20	-6277.322	1684.982	-1.13	0.258
Primary medical and health institutions	-1985.34	2028.53	-5961.2588	1990.5788	-0.98	0.328
Maternal and child health hospital	0.00	.	.	.	.	.

Note: *F* = 264.780. ^∗∗∗^*P* < 0.001.

## Data Availability

The data used to support the findings of this study are available from the corresponding author upon request.
